# Biomonitoring of Metals in Children Living in an Urban Area and Close to Waste Incinerators

**DOI:** 10.3390/ijerph17061919

**Published:** 2020-03-16

**Authors:** Agostino Di Ciaula, Patrizia Gentilini, Giusy Diella, Marco Lopuzzo, Ruggero Ridolfi

**Affiliations:** 1 Division of Internal Medicine, Hospital of Bisceglie (ASL BAT), 76011 Bisceglie, Italy; 2 International Society of Doctors for Environment (ISDE), 52100 Arezzo, Italy; patrizia.gentilini@villapacinotti.it (P.G.); ruggero.ridolfi@gmail.com (R.R.); 3 Clinica Medica “A. Murri”, Department of Biomedical Sciences and Human Oncology, University of Bari “Aldo Moro”, 70124 Bari, Italy; 4 Department of Biomedical Science and Human Oncology, University of Study of Bari “Aldo Moro”, 70124 Bari, Italy; giusy.diella@uniba.it (G.D.); marcolopuzzo@gmail.com (M.L.)

**Keywords:** metals, children, toenails, biomonitoring, waste, incinerators

## Abstract

The impact of waste incinerators is usually examined by measuring environmental pollutants. Biomonitoring has been limited, until now, to few metals and to adults. We explored accumulation of a comprehensive panel of metals in children free-living in an urban area hosting two waste incinerators. Children were divided by georeferentiation in exposed and control groups, and toenail concentrations of 23 metals were thereafter assessed. The percentage of children having toenail metal concentrations above the limit of detection was higher in exposed children than in controls for Al, Ba, Mn, Cu, and V. Exposed children had higher absolute concentrations of Ba, Mn, Cu, and V, as compared with those living in the reference area. The Tobit regression identified living in the exposed area as a significant predictor of Ba, Ni, Cu, Mn, and V concentrations, after adjusting for covariates. The concentrations of Ba, Mn, Ni, and Cu correlated with each other, suggesting a possible common source of emission. Exposure to emissions derived from waste incinerators in an urban setting can lead to body accumulation of specific metals in children. Toenail metal concentration should be considered a noninvasive and adequate biomonitoring tool and an early warning indicator which should integrate the environmental monitoring of pollutants.

## 1. Introduction

Waste incineration in industrial plants generates bottom and fly ashes, which are released into atmosphere after appropriate purification. This procedure, however, does not completely remove toxic chemicals from the emissions. Heavy metals (manganese, lead, cadmium, copper, nickel, mercury, thallium, and vanadium in particular) cause concerns for public health [1,2,3,4,5]. Fly ash emitted from waste incinerators contains large amounts of metals, leading to potential ecological risk [6,7] also due to a progressive accumulation in surrounding soils [2,8]. A study investigating heavy metals in fly ash from 15 municipal solid waste incinerators showed that metals could easily leach out, mainly due to the high content of acid soluble fraction and reducible fraction. This might generate elevated environmental risk [7]. Metals are abundantly present in particulate matter produced by waste incinerators (mainly fine and ultrafine particles, PM0.2–2.5), with dominant presence of vanadium, nickel, copper, zinc, cadmium, and lead in fine particles and with magnesium, aluminum and thallium in coarse particles [5]. Fine particles emitted from waste incinerators have elevated content of heavy metals and are more cytotoxic than those emitted from biomass incineration [9], thus contributing to human toxicity [10].

Metals can enter the human body through different routes such as dermal contact, inhalation, and ingestion [11,12]. Children are particularly vulnerable, in terms of biological effects, when exposed to metal pollution [13,14,15,16,17], mainly due to oxidative damage following chronic exposure [18,19]. In pediatric age, the body burden of metals has been linked with a number of pathologic conditions including nononcologic diseases (i.e., altered growth and development [20], obesity [21,22], and neurologic [23,24,25], cognitive [26], and respiratory [19,27] disorders) and cancer [28,29,30].

A recent study characterizing the distribution of heavy metals in ambient air particles (PM1, PM2.5, PM10) emitted from a municipal waste incinerator, indicated that children living close to this industrial plant had a high noncarcinogenic risk and a high lifetime carcinogenic risk following exposure to toxic metals bound to the emitted particles [28]. Several studies explored the concentration of heavy metals (mainly lead, cadmium, mercury, nickel, and chromium) in adults exposed to emissions from waste incinerators [31,32,33,34,35,36]. However, in the majority of cases a limited number of metals have been considered, and the sampling procedures were on blood and/or urine, thus mainly representing short- rather than long-term exposure [37,38,39,40]. Similarly, previous biomonitoring studies in exposed children only determined the body burden of few trace elements (mainly manganese [3], chromium, lead, and cadmium [41,42,43]), not considering the wide panel of metals [1,5] emitted by waste incinerators. 

Thus, studies investigating the long-term accumulation of multiple metals in children living close to waste incinerators are still lacking. Furthermore, noninvasive biomonitoring tools able to determine, in this age class, the health risk deriving from the discharge of hazardous pollutants into the environment are strongly needed. In fact, human biomonitoring has been proposed as more useful to assess possible health effects than environmental monitoring [44,45]. In this respect, human nails have been frequently employed for the assessment of metal exposure of various origin [46], have been used in pediatric age [47,48,49,50,51], and have been indicated as suitable indicators of long-term exposures [52,53].

## 2. Methods

### 2.1. Study Design

The aim of the present study was to measure the body burden of a wide panel of metals (23 different elements, see Section 2.4) in children living in an urban setting, at different distances from two waste incinerators. According to previous evidence, the concentration of metals in toenails was employed as an indicator of chronic environmental exposure [37,38,39,52,53], adjusting results for possible confounders.

### 2.2. Study Population and Area

A public campaign served to explain the aims of the study. Subsequently, a total of 220 children (128 males, age range 6–9 years) were enrolled in the city of Forlì (Emilia-Romagna region, Northern Italy, 117,946 residents in 2017) from December 2016 to March 2017, after parents signed informed consent. Children also agreed to participate as volunteers.

Inclusion criteria were living at the same address in the last 6 months before enrollment, and the presence of a signed informed consent. 

Subjects with previously known diseases were excluded from the study.

In the urban study area, two incinerators are located about 200 m from each other: a municipal solid waste incinerator (total capacity 100,000 Nm^3^/h), and a hospital waste incinerator (total capacity of 21,500 Nm^3^/h). Besides these two plants, according to the official emission inventories, the remaining sources of air pollution in the explored area are vehicular traffic (urban traffic, two major roadways) and domestic heating during cold season.

**Figure 1:**
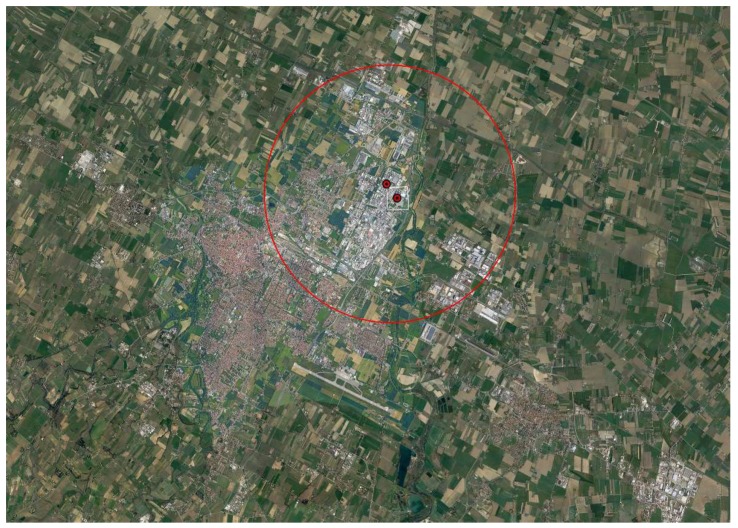
Study area around incinerators (filled circles), in the city of Forlì (Emilia-Romagna, Northern Italy). Exposed subjects considered were 62 children living within a 3 km radius circle around the two incinerators, with the circle centered in the middle of the distance between the two plants. A total of 158 enrolled children were residents in the remaining city areas (reference area).

All enrolled children were georeferentiated. According to previous studies [28,33,41,54,55,56,57] and to results from a dispersion model specifically assessed for the two incinerators [54], exposed subjects were considered those living within a 3 km radius circle around the two plants, with the circle centered in the middle distance between the two (Figure 1). 

Subjects in the reference area (controls) were the residents living outside this circle.

The Romagna Ethical Committee (CEROM) approved the study protocol. The initiative was entirely self-financed with popular events for fundraising or voluntary donations. Written informed consent was signed by both parents.

### 2.3. Assessment of Potential Confounders

A questionnaire served to explore further possible environmental conditions or personal behaviors able to influence the concentration of metals in toenails. Covariates included residential proximity (i.e., less than 300 m) to busy roads, previous orthodontic treatments, regular practice of outdoor sports, hobbies involving the use of chemicals, exposure to passive smoke, and regular consumption of locally grown vegetables. The questionnaire was administered to parents for self-compilation. 

### 2.4. Nail collection, Sample Preparation, and Analysis

Toenails were selected for sampling as preferential to fingernails due to a minor risk of external contamination [58]. The procedures for toenail collection, sample preparation, and analysis have been extensively employed in previous studies [47,48,59,60,61,62,63,64,65,66,67]. 

Toenails were clipped using ceramic blade to avoid possible contamination. Samples were thereafter stored in a 10 mL polypropylene tube for subsequent analysis, and scissors were cleaned with a light-acid solution. Toenails were examined according to a standardized technique [68]. Briefly, samples were immersed in a 70% ethanol solution without stirring or sonication for a period of 10 min, to reduce the risk of microbiological contamination. Exogenous impurities were removed by a multistep washing procedure with acetone and Milli-Q purified water, and the cleaned samples were kept at room temperature for a period from 24 to 48 h for drying. 

The dry samples were weighed, and the concentration of 23 elements (Aluminum (Al), Antimony (Sb), Arsenic (As), Barium (Ba), Beryllium (Be), Boron (B), Cadmium (Cd), Chromium (Cr), Cobalt (Co), Iron (Fe), Manganese (Mn), Mercury (Hg), Molybdenum (Mo), Nickel (Ni), Lead (Pb), Copper (Cu), Selenium (Se), Thallium (Tl), Thorium (Th), Tungsten (W), Uranium (U), Vanadium (V) and Zinc (Zn)) was subsequently calculated, using inductively coupled plasma mass spectrometry (ICP-MS) and the EPA 6020A 2007 method.

### 2.5. Statistical Analysis

Frequencies of categorical variables and means and standard errors of continuous variables were calculated. The Wilcoxon test or the chi-squared test were employed to compare differences among groups. Correlations were tested using the Spearman’s rank correlation coefficient. Tobit regression models were employed to examine the association between the concentration of metals and potential influencing factors. Tobit regression was also used to accommodate the left-censored nature of values, due to the presence of samples with metal concentration below the limit of detection [69]. Metal concentrations were log-transformed to meet the normal assumption [70]. *P* values < 0.05 were considered statistically significant.

Analyses were performed using R software version 3.5.1 (R Project for Statistical Computing, available from https://www.r-project.org/).

## 3. Results

According to georeferentiation, totals of 62 and 158 children were residents within 3 km from the incinerators (exposed area) and in the control area, respectively.

**Figure 2:**
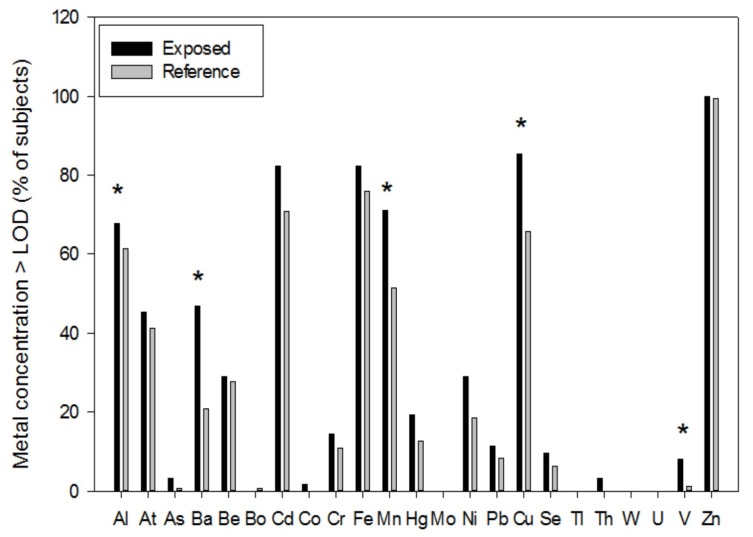
Proportion of children with metal concentration in toenails above the limit of detection (LOD). Children living within a 3 km radius from the two incinerators were considered exposed (n = 62). Children in the reference area (n = 158) were those living in the remaining city areas. Asterisks indicate *p* < 0.01 (chi-squared test).

The concentrations of Mo, Tl, W, and U were lower than the limit of detection (LOD) in all collected toenail samples, irrespective of residence (Table 1 and Figure 2). The concentrations of As, Co, and Th were above the LOD in three (4.8%), one (1.6%) and two (3.2%) subjects living in the exposed area, respectively, but in none of those living in the reference areas. Conversely, Bo was only measurable in one subject living in the reference area.

As shown in Figure 2, the percentage of children with toenail metal concentrations above the LOD tended to be higher in those living in the exposed than in those living in the reference area in all cases, with significantly higher proportions for Al (67.7% vs. 61.4%, respectively), Ba (46.8% vs. 20.9%), Mn (71% vs. 51.3%), Cu (85.5% vs. 65.8%) and V (8.1% vs. 1.3%).

Table 1 shows the absolute concentrations of metals measured in the two groups of children. Children living within 3 km around the incineration plants had significantly higher concentrations of Ba, Mn, Cu, and V, as compared with those living in reference area. On average, the concentrations of these metals were, respectively, 5.5, 1.8, 1.3 and 9.5 times higher in children living in the exposed area than those in the control area.

According to results of the Tobit regression (Table 2), living in the exposed area was a significant predictor of Ba, Ni, Cu, Mn, and V concentrations, after adjusting for covariates. The analysis of covariates also showed influencing effects of previous orthodontic treatments on Ba and Cu concentrations and of exposure to passive smoke on Ba concentrations. However, the proportions of children with previous orthodontic treatments (9.7% in exposed, 10.1% in reference area, *p* = NS) or exposed to passive smoke (3.2% in exposed, 6.3% in reference area, *p* = NS) were similar in the two groups of children.

Considering the whole group of subjects, the Spearman’s correlation matrix showed that Ba, Mn, Ni, and Cu (but not V) were correlated with each other, suggesting the possibility of a common source of emission (Table 3).

## 4. Discussion

Results from the present study show for the first time an increased body burden of specific metals in children free-living in an urban area and exposed to emissions from waste incinerators, as compared with controls. 

We used toenails as a biomarker of exposure to metals. Metals bind keratin proteins maintaining a stable concentration over time, independently from changes in metabolic activities [38,39]. The slow rate of growth of toenails (on average 1.62 mm/month) [37] allows to evaluate longer term exposure [37], as compared with blood or urine [38,39,40]. Few studies evaluated the correlation between the concentration of metals in nails and in other biological matrices, with variable results [40] probably due to the different time windows that can be explored using nails (6–12 months earlier [37,40,67,71,72]), blood (2–3 h [73]), and urine (3–4 days [74]). Positive correlations have been documented between concentration in toenails, urine, and blood in the case of Mn [75] which, in the present study, has been found in higher concentration in exposed children than in controls. Of note, positive correlations have been demonstrated between the concentrations of metals in toenails and in environmental matrices such as dust [61,71,76,77], soil [61,63,77,78], and water [63,79], confirming the adequacy of toenail as a biomarker of environmental exposure.

In exposed subjects, we found metals that, conversely, were in all cases below the LOD (As, Co, and Th) or were present in significantly lower concentrations (in particular Ba, Mn, Cu, and V) in children living in the reference area. Living within a 3 km circle from waste incinerators was a significant predictor of Ba, Ni, Cu, Mn, and V concentrations, after adjusting for covariates. The presence, in our study, of a correlation between the concentrations of these metals (with the exception of V) points to a probable common source of exposure. A recent health-risk assessment study indicated that ambient air around 3 km from a municipal waste incinerator had more PM1, PM2.5, and PM10 particles than general nonpolluted air [28]. The cited study also showed high noncarcinogenic risk and lifetime carcinogenic risk for children, derived from incinerator-emitted particle-bound toxic metals [28]. 

Our results are in line with a previous study determining air pollutants collected downwind from an Italian incinerator and showing that Mn, Cu, Ba, and V were among metals with the highest concentrations in both the fine and coarse fractions of the particulate matter [1]. In a study assessing the short-term oxidative potential of urban particulate matter in adult nonsmoking volunteers, several metals present in coarse, fine, and ultrafine PM (including Ba, Cu, Ni, and V) were significantly associated with increased levels of biomarkers of systemic inflammation, oxidative stress, and neural function. Ba, in particular, induced a significant increment (+11% at 1 h, +14% at 21 h postexposure) of L1(UCHL1) (traumatic brain injury marker ubiquitin C-terminal hydrolase L1); Cu exposure increased (+14% at 1 h) levels of the DNA oxidation marker 8-hydroxy-deoxy-guanosine; urinary cortisol increased by 88% after exposure to V, and the blood inflammatory marker VEGF (vascular endothelia growth factor) increased by 5.3% 1 h after Ni exposure [80].

Toenail concentration of Mn has been frequently studied both in children [47,48,49,50] and in adults [40], with an LOD ranging from 0.001 [81] to 0.33 μg/g [66] and values usually below 10 μg/g [40]. The highest Mn toenail concentrations have been found in subjects living near a highly industrialized city in Pakistan (average value 52.1 μg/g) [82] and in highly polluted areas in Cambodia (average concentration 43.9 μg/g) [66]. In our study, the average Mn concentration recorded in toenails from exposed children (4.4 μg/g) was slightly higher than that previously reported in pediatric age (3.57 μg/g, weighted means) in an analysis of pooled literature [47].

In Brazilian children aged 11–16 years and living in an urban area, fingernail metal concentrations are linked with the degree of urbanization (i.e., population density) and with the extent of vehicular traffic. This explains about half (50.8%) of the variance in metal concentration. In the cited study, the average Mn nail concentration measured in subjects living in the area with the highest population density was 1.3 μg/g, a value about 3.3 times lower than the mean Mn concentration detected in our series of exposed children [48]. This difference could be due, at least in part, to the coexisting exposure in our series of exposed children to vehicular traffic and industrial pollution. In fact, the average Mn nail concentration measured in our study in children living in the reference area (2.47 μg/g) and mainly exposed to vehicular traffic was close to that reported in Brazilian children.

According to a previous observation, urinary concentrations of Mn are inversely related to the distance of residence from a municipal solid waste incinerator, and are directly linked with the exposure to particulate matter [31]. Mn was present at the highest level among heavy metals in particulate matter collected downwind of an Italian incinerator [1]. Mn has also been described as the metal with the highest concentration in soil [2,83] and with the second highest concentration in air (following Cu [83] or Pb [28]) around a solid waste incinerator.

Inhaled Mn can cross the blood–brain barrier and can enter the brain through axonal transport from the olfactory bulb to the cerebral cortex [84]. Children might be particularly at risk from Mn inhalation. In children aged 7–9 years living in East Liverpool (Ohio), a site with a hazardous waste incinerator and a manganese processor, a link has been shown between blood/hair Mn levels and neurological effects (altered IQ score) after adjusting for potential confounders [3]. 

In our series of enrolled children, the average Mn concentration in toenails from exposed subjects (4.4 μg/g) was 3 times higher than that measured (1.43 μg/g) in toenails from 225 school-age children (7–12 years) living in a Brazilian industrial region. In this group of subjects, a relationship has been demonstrated between high toenail Mn concentrations and the increased risk of intellectual deficit linked to Pb exposure, although the exposure was low (only 1.8% of children were above the CDC reference value of 5 µg/dL) [49]. A study assessing Mn accumulation in children aged 7–12 years and living near a ferro-manganese alloy plant indicated toenail Mn as a biomarker of environmental exposure, associating the burden of this metal in exposed subjects with disrupting neurobehavior. Of note, in exposed children, the median Mn toenail concentration recorded in the cited study was about 5 times lower than the mean value (0.84 μg/g) observed in our study [50].

Studies exploring the specific concentrations of heavy metals in air samples collected around a Spanish municipal solid waste incinerator showed that the highest concentration was registered for Cu [83,85]. Previous studies assessed nail Cu concentrations both in adults [40,86] and in children [47], indicating an LOD ranging, for this metal, from 0.009 to 0.12 μg/g, with values usually below 10 μg/g [40]. The highest Cu concentration in nails (average value 26.2 μg/g) has been recorded in subjects living in rural areas near a highly industrialized city in Pakistan [82]. The average Cu toenail concentration recorded in our series of exposed children (6.34 μg/g) was slightly higher than that (5.66 μg/g) measured in nails from Arab-American children living in a highly industrialized US area [47].

An increased Cu body burden has been related with increased oxidative stress secondary to the reduction of antioxidant enzyme activity and the generation of reactive oxygen species (ROS). These events are able to promote DNA damage, favoring the onset of cancer [87]. A recent study compared metal concentrations in nails from adults with non-Hodgkin or Hodgkin lymphoma, showing higher Cu levels in both groups of patients as compared with healthy controls. In the cited study, the mean nail Cu concentration in controls (4.8 μg/g) was very similar to that observed in our series of children living in the control area (4.74 μg/g), and Cu concentrations in nail samples from lymphoma patients (7.36 and 7.76 μg/g in non-Hodgkin and Hodgkin lymphoma, respectively) were only slightly higher that the average Cu concentration recorded, in our study, in exposed children (6.34 μg/g) [86].

Of note, children’s exposure to Cu has also been linked with nononcologic conditions such as neurologic disorders [23,24,25] and obesity [22]. Significantly higher blood Cu concentrations have been found in obese children, as compared with healthy controls [88,89]. Additionally, a large cross-sectional survey on US children and adolescents demonstrated a strong association between the highest quartile of blood Cu concentration and obesity [22].

Ba is not essential in human nutrition, but, as mainly suggested by animal studies, health effects secondary to chronic Ba exposure are possible in humans, although results from epidemiologic studies are still scarce [90], as are biomonitoring reports [91]. The main routes of nonoccupational human exposure to Ba are the ingestion of contaminated food and/or water [90]. However, this metal is also frequently detected in particulate matter produced by several industrial combustion processes, including waste incineration [1,90].

In a group of 126 healthy Brazilian children living in an urban area (Porto Alegre) [51], mean Ba concentration in nails (5.6 μg/g) was 2.6-fold higher than that observed, in our study, in children living in the control area (2.15 μg/g), but 2-fold lower than that recorded in our exposed children (11.9 μg/g). 

The average concentration of Ba observed in toenails from our series of exposed children was also about 9 times higher than the average Ba nail concentration measured in Arab-American children living in a highly industrialized US area (1.28 μg/g) [47] and 3.7 times higher than that reported in a series of 145 adults (3.21 μg/g), in whom a significant association between Ba levels in toenails and hearing loss at 8 kHz and 12 kHz was demonstrated after adjustment for sex, age, body mass index, and smoking [92].

Recently, Ba exposure during pregnancy (assessed by measuring Ba concentrations in maternal hair and in fetal placenta) has been dose-dependently linked with the risk of congenital heart defects in offspring, underlying health hazards deriving from prenatal and transplacental exposure to this metal [93]. Furthermore, data from the National Health and Nutrition Examination Survey (NHANES 1999-2011) found, in a large cohort of US children aged 6–19 years, a strong association between Ba exposure (urinary Ba concentration) and obesity [21].

Several studies measured the concentration of V in nails [40,47,48,81,94,95], reporting an LOD of 0.001 μg/g [81], an inverse relation with age [47,94,95], and values generally lower than 1 μg/g [40]. This was also the case of toenail V concentration measured in the exposed children from our study (0.19 μg/g). This concentration, however, was higher (about double) than that recorded in nails from Arab-American children living in a highly industrialized US area (0.09 μg/g) [47], in a series of Brazilian children living in an urban area (0.08 μg/g) [48], and as compared with the average concentration (0.11 μg/g) derived from pooled literature values in pediatric age [47].

In the present study, toenail concentration of V was higher in children living in the exposed area than in those in the control area. However, there was no relationship between toenail concentration of V and concentrations of Ba, Mn, Ni, and Cu that, conversely, were correlated with each other. This result could be due to a local source of anthropogenic emission of V different from the two incinerator plants. On the other hand, it is also possible that the same plants generate V, but through combustion processes not involving solid waste. In fact, air concentrations of V have been used as an indicator of emissions from oil combustion [96,97,98], and it has been suggested that burning waste oil in incinerators or using oil for providing power in incinerator plants can generate V emissions [98]. Vanadium has been measured in air samples around a Spanish incinerator [85], and a cross-sectional study assessing metal concentrations in spot urine samples from subjects living within 4 km from an Italian incinerator showed, in exposed subjects, V levels higher than the reference value for the Italian population [32].

According to our results, living in the exposed area was a significant predictor of toenail Ni concentrations, which were related with toenail concentrations of Ba, Mn, and Cu. Data also showed a trend towards an increased toenail concentration of Ni in children living in the exposed area compared to those in the control area.

Ni has been detected in both air and soil samples collected around a Spanish municipal solid waste incinerator [85], and a study in Taiwan showed that the burden of this metal in the local airborne particles was highly influenced by the stack emission of the local incinerator [4]. Additionally, a study analyzing samples of particulate matter collected in proximity of a Chinese municipal solid waste incinerator described fine particles as dominant, as compared with coarse and ultrafine particles, and anthropogenic metal elements (including Ni, Cu, V) predominantly concentrated in fine particles [5].

The average toenail concentration of Ni measured, in our study, in exposed children (2.23 μg/g), was slightly higher than that (1.8 μg/g) found in Brazilian children living in an urban area with high population density [48], but much lower than the mean concentration detected in Arab-American children living in an urban setting in a highly industrialized area (45.18 μg/g) [47].

In children, the exposure to Ni in particulate matter is negatively associated with indices of lung function. Ni vehiculated by PM10, in particular, has been linked with decrements in forced expiratory volume in the first second [99] and, according to data from school children living in an e-waste recycling area, the accumulation of Ni in serum could generate oxidative damage and decreased pulmonary function [19]. A recent study determining the concentrations of Ni in hair of pregnant women and in fetal placental tissues demonstrated a possible effect of Ni exposure in increasing the occurrence of congenital heart defects in offspring [100]. Finally, Ni is a IARC Group 1 carcinogen, and a possible relationship has been suggested between urinary Ni levels and childhood acute leukemia, secondary to oxidative DNA damage [101].

A recent report showed increased blood levels of heavy metals (Cr, Pb, Cd), DNA damage and epigenetic changes (altered DNA methylation) in school age children living within 3 km around a Chinese waste incinerator [41]. This study confirmed previous evidence reporting higher Pb and Cd concentrations in blood samples from adolescents living near a Belgian incinerator than in controls [42]. Unfortunately, however, in both these reports, information on body levels of other metals are lacking. 

In our series, children living in exposed or in control area showed similar toenail concentrations of Cd, Cr, and Pb. However, the proportion of subjects with concentration of these metals below the LOD tended to be higher in the control area. Our results are in line with two previous works reporting, in adults, no associations between living near a municipal solid waste incinerator and blood Pb and Cd levels [34,35]. Conversely, higher blood concentrations of Cr and Pb have been reported in adults living close to Chinese waste incinerators than in controls, with vegetable ingestion being the main contributor to the total average daily dose of these metals, as compared with Mn [33]. 

Thus, the possibility exists that site-specific exposure pathways (mainly dependent on dietary habits in rural areas) could influence the internal metal levels in exposed subjects, with consumption of local vegetables grown near incinerators being at risk for specific (i.e., Cr, Pb) body metal accumulation. In fact, in our series of enrolled children (all living in an urban area), consumption of locally grown vegetables was scarce and not linked with metal concentration. In this case, inhalation, rather than ingestion, could be the main exposure route.

In the present study, analysis of covariates suggested a possible influencing effect of previous orthodontic treatments and passive smoke on nail concentration of Ba (both factors) and Cu (only passive smoke). However, the role of these confounders seems to be limited, since living in the exposed area was a significant predictor of Ba, Ni, Cu, Mn, and V nail concentrations after adjusting for all considered covariates. Furthermore, no difference was evident in the distribution of subjects with previous orthodontic treatments and/or passive smoke in the two groups of explored children.

A previous longitudinal study based on dispersion modeling for exposure assessment explored health outcomes in a large cohort of subjects living in the same area examined in our study (3.5 km around the two incinerators of Forlì). Results showed significant associations between increasing heavy metal exposure and cause-specific mortality: colon cancer in men; all cancer sites, stomach, colon, liver and breast cancer in women; and excess of soft tissue sarcoma in the two sexes combined [54]. These findings point to the existence of an increased health risk in the same urban area in which results from our study have shown a greater internal accumulation of metals in exposed children, as compared with those living in the reference area. 

Metals should be considered an indicator of exposure to a complex combination of pollutants generated from waste combustion, including gaseous pollutants, persistent organic pollutants, and a number of other toxic chemicals vehiculated by particulate matter. From this point of view, it should be underlined that cumulative exposure to complex mixtures of chemicals of industrial origin may generate synergistic effects on health [102]. Moreover, possible interactions between multiple and heterogeneous exposures (i.e., industrial pollution, vehicular traffic, contaminated water/food), should overcome the single-pollutant approach with the measurement of the absorbed internal dose of multiple pollutants (the exposome [103]).

Finally, some metals are characterized by a linear dose-response with low-dose effects and no threshold (i.e., Cu, Cd) or by a nonlinear dose-response with low-dose effects (i.e., Ni) [104]. These aspects also generate concern if metals are released in the environment at low concentrations. 

Taken together, all these aspects amplify the possibility of health risk in pediatric age, also considering that children are more vulnerable to environmental toxins and have significantly more time, as compared with adults, for developing chronic effects of protracted environmental exposures, including both cancer and noncommunicable diseases.

## 5. Conclusions

The release of metals from waste incinerators located in an urban area can contribute to human toxicity following chronic exposure, in particular in children. 

The present study employed the concentration of metals in toenails as an expression of long-term body accumulation of a wide panel of metals, demonstrating, in children living close to waste incinerators, an increased concentration of specific metals (in particular Ba, Mn, Cu, and V) potentially leading to an increased health risk.

Measuring the concentration of metals in toenails should be considered a noninvasive and adequate biomonitoring tool and an early warning indicator, which could allow a more realistic and comprehensive analysis of risk assessment as compared with the simple monitoring of environmental pollutants.

## Figures and Tables

**Table 1:** Absolute concentrations of metals (μg/g) in toenails from children living within a 3 km radius circle around the two incinerators (exposed area) or in the reference area.

Metal	Exposed Area (n = 62)	Reference Area (n = 158)	*p*
Al	166.48 ± 50.42	103.24 ± 11.01	NS
At	0.07 ± 0.02	0.10 ± 0.02	NS
As	0.01 ± 0.01	0.00	NS
Ba	11.95 ± 9.01	2.15 ± 0.87	<0.0002
Be	0.03 ± 0.01	0.03 ± 0.005	NS
Bo	0.00	0.15 ± 0.15	NS
Cd	0.03 ± 0.004	0.07 ± 0.02	NS
Co	0.04 ± 0.04	0.00	NS
Cr	4.82 ± 3.88	1.28 ± 0.44	NS
Fe	360.08 ± 126.57	164.49 ± 21.06	NS
Mn	4.40 ± 1.23	2.47 ± 0.35	<0.05
Hg	0.05 ± 0.01	0.06 ± 0.02	NS
Mo	0.00	0.00	NS
Ni	2.23 ± 1.51	0.43 ± 0.18	NS
Pb	0.32 ± 0.13	0.95 ± 0.47	NS
Cu	6.34 ± 0.70	4.74 ± 0.36	<0.05
Se	0.01 ± 0.005	0.01 ± 0.003	NS
Tl	0.00	0.00	NS
Th	0.01 ± 0.01	0.00	NS
W	0.00	0.00	NS
U	0.00	0.00	NS
V	0.19 ± 0.11	0.02 ± 0.02	<0.02
Zn	96.27 ± 9.42	95.30 ± 3.09	NS

Legend: values are expressed as means and standard errors. NS, not significant.

**Table 2:** Results of Tobit regression model on metal concentrations in toenails from children living within a 3 km radius circle around the two incinerators (exposed area) or in the reference area, and the effect of covariates.

	Ba	Ni	Cu	Mn	V
Exposed vs. Reference	0.76 *** (0.4 to 1.1)	0.31 * (0.05 to 0.6)	0.22 ** (0.06 to 0.4)	0.2 * (0.06 to 0.4)	1.08 * (0.2 to 2.0)
Residential proximity to busy roads	−0.13 (−0.5 to 0.2)	−0.18 (−0.4 to 0.05)	0.09 (−0.03 to 0.2)	−0.1 (−0.3 to 0.01)	−0.1 (−0.8 to 0.5)
Orthodontic treatments	−0.87 * (−1.6 to −0.2)	−0.08 (−0.5 to 0.3)	0.3 * (0.04 to 0.5)	−0.05 (−0.3 to 0.2)	0.8 (−0.1 to 1.7)
Outdoor sports	0.13 (−0.2 to 0.5)	0.08 (−0.2 to 0.3)	0.006 (−0.1 to 0.2)	0.1 (−0.03 to 0.3)	−0.3 (−1.0 to 0.4)
Hobbies involving chemicals	0.08 (−0.2 to 0.4)	−0.07 (−0.3 to 0.2)	−0.1 (−0.3 to 0.006)	0.06 (−0.09 to 0.2)	−0.2 (−0.9 to 0.5)
Passive smoke	0.8 * (0.3 to 1.4)	0.36 (−0.07 to 0.8)	0.09 (−0.2 to 0.4)	0.2 (−0.2 to 0.5)	1.0 (−0.2 to 2.2)
Consumption of locally grown vegetables	0.1 (−0.06 to 0.3)	0.04 (−0.08 to 0.2)	−0.008 (−0.08 to 0.07)	0.09 (0.01 to 0.2)	0.005 (−0.4 to 0.4)
*Constant*	0.05 (−0.1 to 0.2)	−0.2 (−0.5 to −0.1)	−0.59 (−0.7 to −0.5)	−0.5 (−0.6 to −0.4)	−0.04 (−0.6 to 0.5)

Legend: only significant results (metal concentration) are presented. Metal concentrations were log-transformed to meet the normal assumption. Results (β coefficients and 95% confidence intervals) have been adjusted for covariates and consider the left-censored data present in metals distribution. * *p* < 0.05, ** *p* < 0.02, *** *p* < 0.001.

**Table 3:** Spearman’s correlation matrix considering the toenail concentrations of Ba, Mn, Ni, Cu, and V in the whole group of enrolled children (n = 220).

	**Ba**	**Mn**	**Ni**	**Cu**	**V**
**Ba**	-	0.45	0.36	0.23	0.13
	-	***<0.000001***	***<0.000001***	***0.0006***	*0.059*
**Mn**	0.45	-	0.36	0.37	0.09
	*<0.000001*	-	***<0.000001***	***<0.000001***	*0.17*
**Ni**	0.36	0.36	-	0.23	0.09
	*<0.000001*	***<0.000001***	-	***0.0006***	*0.18*
**Cu**	0.23	0.37	0.23	-	0.02
	*0.0006*	***<0.000001***	***0.0006***	-	*0.82*
**V**	0.13	0.09	0.09	0.02	-
	*0.059*	*0.16*	*0.18*	*0.82*	-

Legend: data are Spearman correlation coefficients (rho, normal text) and *p*-values (in italic). Significant *p*-values are marked in bold.
